# Prospective evaluation of the effect of adherent perinephric fat on outcomes of robotic assisted partial nephrectomy following elimination of the learning curve

**DOI:** 10.1590/S1677-5538.IBJU.2019.0097

**Published:** 2019-12-17

**Authors:** Ashley M. Shumate, Grayson Roth, Colleen T. Ball, David D. Thiel

**Affiliations:** 1 Department of Urology, Mayo Clinic, Jacksonville, FL, USA; 2 Department of Health Sciences Research, Mayo Clinic, Jacksonville, FL, USA; 3 Division of Biomedical Statistics and Informatics, Mayo Clinic, FL, USA

**Keywords:** Carcinoma, Renal Cell, Robotic Surgical Procedures, Nephrectomy

## Abstract

**Purpose:**

To prospectively evaluate the association of adherent perinephric fat (APF) on perioperative outcomes of robotic-assisted partial nephrectomy (RAPN) following elimination of the surgical learning curve.

**Materials and Methods:**

305 consecutive RAPNs performed by a single experienced surgeon were analyzed. The first 100 RAPNs were considered the learning curve and therefore excluded. APF was defined as the necessity of subcapsular renal dissection to mobilize the tumor from surrounding perinephric fat. Perioperative outcomes were evaluated including operative time, warm ischemia time (WIT), postoperative complications, length of stay, margins, ischemia, and complications score (MIC), estimated blood loss (EBL), and change in pre-operative to postoperative day 1 (POD 1) laboratory values. After correction for multiple comparisons, P values ≤0.0045 were considered statistically significant but associations with P values ≤0.05 were also mentioned in the study results.

**Results:**

Fifty-eight (28.3%) patients had APF. Patients with APF had longer operative times compared to those without APF (median, 213 vs. 192 minutes, P <0.001). There was some evidence of higher increase in change in creatinine from preoperative to POD 1 among those with APF compared to those without APF, although this was not statistically significant (median, 0.2 vs. 0.1mg/dL, P=0.03). There were no other statistically significant associations between presence of APF and perioperative outcomes.

**Conclusions:**

APF is associated with increased operative time but no change in other perioperative outcomes. Surgeon experience does not affect perioperative outcomes associated with APF.

## INTRODUCTION

The learning curve of robotic assisted partial nephrectomy (RAPN) is steep. For surgeons already experienced with laparoscopic partial nephrectomy, the true learning curve has been proposed to be up to 65 cases, depending on the definition of proficiency (1). Adherent perinephric fat (APF), colloquially known as “sticky fat” can frustrate surgeons and complicate RAPN. Previous studies have shown APF to increase both operative time (2, 3) and estimated blood loss (EBL) during RAPN (4, 5). There has also been an association of APF with increase in transfusion rate and conversion to either open or radical nephrectomy (6). However, to our knowledge, no study has examined the effect of surgeon experience and the surgical learning curve on the adverse outcomes associated with APF during RAPN. We previously demonstrated that APF prolonged operating room time of RAPN in a prospective evaluation of 100 patients (2). We examined if surgical experience eliminated the effect of APF on RAPN outcomes.

## MATERIALS AND METHODS

### Data Collection

All consenting patients undergoing surgery for a suspicious renal mass at our institution are prospectively included in an IRB-approved registry. We prospectively collect clinical, pathologic, and demographic data, as well as biological specimens (tumor tissue, blood, urine). At time of study, our registry included 305 patients who underwent RAPN by a single surgeon. To eliminate outcomes associated with the learning curve, the first 100 RAPN were excluded from our analysis. Adherent perinephric fat was recorded by the operative surgeon and defined as the necessity of subcapsular dissection to isolate the tumor from its surrounding perinephric fat (2).

We collected patient characteristics at baseline (age, sex, body mass index [BMI], preoperative creatinine, preoperative hemoglobin, preoperative estimated glomerular filtration rate [eGFR], hypertension, cardiovascular disease, diabetes, and history of smoking), tumor information (size of renal mass, R.E.N.A.L. nephrometry score (7), and Mayo Adhesive Probability [MAP] score (8)), operative information (EBL, operative time, warm ischemia time (WIT), conversion to open partial nephrectomy or laparoscopic nephrectomy, and surgical margins [positive or negative]) and postoperative information (hemoglobin at postoperative day [POD 1], creatinine at POD 1, eGFR at POD 1, postoperative complication grade I-V, and length of hospital stay [LOS]). Postoperative complications were catergorized by Clavien-Dindo grade (9). We additionally defined margins, ischemia, and complications (MIC) as those who had negative surgical margins, a warm ischemia time less than 20 minutes, and no postoperative complications higher than grade II (10). MAP and R.E.N.A.L. scores were calculated according to the algorithms previously described (7, 8). MAP scores of 0-3 were considered low and scores of 4-5 considered high. R.E.N.A.L. scores were grouped 4-6, 7-9, and 10-12, respectively. For patients with multiple renal masses, the size of the largest resected mass was used for analysis. All RAPN were performed in standard fashion as previously described utilizing sliding-clip renorrhaphy (2, 11).

#### Statistical analysis

Associations of perioperative outcomes with presence of APF and high MAP score (>3) were evaluated using the Wilcoxon rank-sum test for continuous outcomes and the Fisher's exact test for categorical outcomes. Associations of perioperative outcomes with BMI and R.E.N.A.L. score were evaluated using the Spearman rank correlation test for continuous outcomes and the Cochran-Armitage trend test for categorical outcomes. To account for the increase in the likelihood of a type I error (i.e., a false-positive finding) secondary to the number of statistical tests performed, we used a Bonferroni correction separately when evaluating associations of perioperative outcomes with APF, BMI, R.E.N.A.L. score, and MAP score. After correction for multiple comparisons, P values ≤0.0045 were considered statistically significant but associations with P values <0.05 were also mentioned in the study results. All analyses were performed using SAS (version 9.4, SAS Institute Inc., Cary, NC).

## RESULTS

Among the 205 patients, 58 (28.3%) had APF detected during RAPN. Patient and renal mass characteristics of the cohort are summarized in Table-1. Comparisons of perioperative outcomes between patients with APF and patients without APF are displayed in Table-2. Patients with APF had longer operative times compared to patients without APF (median, 213 vs. 192 minutes; P <0.001). No other associations between presence of APF and perioperative outcomes were statistically significant after applying the Bonferroni adjustment (P ≤0.0045 considered significant). However, there was some evidence of higher increase in change in creatinine from preoperative to POD 1 among those with APF compared to those without APF (median, 0.2 vs. 0.1mg/dL, P=0.03) but this was not reflected in number of patients with eGFR ≤60mL/min/1.73m^2^. Conversion to laparoscopic nephrectomy was required in 5 cases, none of which were in patients with APF.

**Table 1:** Patient and renal mass characteristics.

Characteristic		All patients (n=205)
Age, years		63 (22, 56, 71, 84)
**Age distribution, n (%)**		
	< 60 years	76 (37.1%)
	60-65 years	34 (16.6%)
	> 65 years	95 (46.3%)
Female sex, n (%)		82 (40.0%)
Body mass index, kg/m^2^		28.7 (16.5, 25.6, 33.0, 60.6)
**Body mass index distribution, n (%)**		
	< 25 kg/m^2^	43 (21.0%)
	25-30 kg/m^2^	78 (38.0%)
	> 30 kg/m^2^	84 (41.0%)
Preoperative creatinine, mg/dL		1.0 (0.5, 0.8, 1.1,3.1)
Preoperative hemoglobin		13.9 (10.7, 12.8, 14.7, 18.0)
Preoperative eGFR < 60 mL/min, n (%)		32 (15.6%)
Hypertension, n (%)		131 (63.9%)
Cardiovascular disease, n (%)		36 (17.6%)
Diabetes, n (%)		41 (20.0%)
History of smoking, n (%)		59 (28.8%)
Size of renal mass, cm		3.1 (1.0, 2.5, 4.2, 10.0)
Adherent perinephric fat, n (%)		58 (28.3%)
RENAL nephrometry score		8 (4, 7, 9, 11)
**RENAL nephrometry score distribution, n (%)**		
	4-6	46 (22.4%)
	7-9	109 (53.2%)
	10-12	50 (24.4%)
Mayo Adhesive Probability score		1 (0, 0, 4, 5)
Mayo Adhesive Probability score > 3, n (%)		59 (28.9%)

Data are given as the median (minimum, 25^th^ percentile, 75^th^ percentile, maximum) or number (percent). Mayo adhesive probability score was not available for 1 patient.

**Table 2:** Comparisons of perioperative outcomes according to presence of adherent perinephric fat.

Perioperative outcome		Adherent Perinephric Fat (n=58)	No Adherent Perinephric Fat (n=147)	P value
Operative time, min		213 (148, 188, 228, 336)	192 (106, 169, 216, 320)	<0.001
Warm ischemia time, min		19 (0, 14, 24, 35)	19 (0, 14, 23, 42)	0.33
Conversion to open or laparoscopic nephrectomy, n (%)		0 (0.0%)	5 (3.4%)	0.32
Estimated blood loss (mL)		400 (300, 300, 500, 1200)	300 (100, 300, 500, 1500)	0.17
Any postoperative complication, grade I-V, n (%)		13 (22.4%)	31 (21.1%)	0.85
Postoperative complication, grade III-V, n (%)		3 (5.2%)	11 (7.5%)	0.76
**Change in laboratory measures (preoperative to POD 1)**				
	Hemoglobin, mg/dL	−2.3 (−5.5, −3.2, −1.7, −0.2)	−2.1 (−11.2, −2.8, −1.6, 0.0)	0.30
	Creatinine, mg/dL	0.2 (−0.3, 0.0, 0.4, 1.2)	0.1 (−0.3, 0.0, 0.3, 1.1)	0.034
eGFR≤60, n (%)		24/47 (51.1%)	47/124 (37.9%)	0.16
Length of hospital stay, days		2 (1, 2, 3, 25)	3 (1, 2, 3, 25)	0.56
Length of hospital stay > 3 days, n (%)		14 (24.1%)	29 (19.7%)	0.57
MIC, n (%)		30 (51.7%)	79 (55.2%)	0.75

**POD =** postoperative day; **eGFR =** estimated glomerular filtration rate; **MIC =** margins, ischemia, and complications.

Data are given as the median (minimum, 25^th^ percentile, 75^th^ percentile, maximum) or number (percent). The change in eGFR from preoperative to POD 1 was reported as the fraction (percent) of patients with an eGFR≤60 at POD 1 among only those who had a preoperative eGFR>60. P values result from the Wilcoxon rank-sum test or the Fisher's exact test. P values ≤ 0.0042 were considered statistically significant after applying a Bonferroni adjustment. Warm ischemia time was not available for 3 patients whose surgery converted to nephrectomy. Laboratory measures at POD 1 were not available for 2 patients. MIC was not available for 4 patients.

Associations of BMI with perioperative outcomes are shown in Table-3. There were no statistically significant associations after adjustment for multiple comparisons or even notable associations of BMI with perioperative outcomes.

**Table 3:** Perioperative outcomes according to body mass index.

Perioperative outcome		BMI <25 kg/m^2^ (n=43)	BMI 25-30 kg/m^2^ (n=78)	BMI >30 kg/m^2^ (n=84)	P value
Operative time, min		191 (125, 159, 210, 240)	198 (106, 175, 227, 279)	196 (116, 175, 224, 336)	0.10
Warm ischemia time, min		18 (0, 13, 22, 29)	19 (0, 14, 24, 42)	19 (0, 14, 24, 34)	0.55
Conversion to open or laparoscopic nephrectomy, n (%)		1 (2.3%)	3 (3.8%)	1 (1.2%)	0.55
Estimate blood loss (mL)		300 (200, 300, 400, 800)	300 (100, 300, 500, 1200)	400 (100, 300, 500, 1500)	0.10
Any postoperative complication, grade I-V, n (%)		13 (30.2%)	16 (20.5%)	15 (17.9%)	0.13
Postoperative complication, grade III-V, n (%)		4 (9.3%)	4 (5.1%)	6 (7.1%)	0.77
**Change in laboratory measures (preoperative to POD 1)**					
	Hemoglobin, mg/dL	−2.2 (−5.8, −3.0, −1.7, −0.2)	−2.2 (−11.2, −3.1, −1.6, 0.0)	−2.1 (−5.0, −2.7, −1.6, −0.5)	0.62
	Creatinine, mg/dL	0.1 (−0.2, 0.0, 0.3, 0.7)	0.2 (−0.3, 0.0, 0.4, 1.2)	0.2 (−0.3, 0.1, 0.4, 1.1)	0.079
	eGFR ≤ 60, n (%)	9/36 (25.0%)	30/65 (46.2%)	32/70 (45.7%)	0.070
Length of hospital stay, days		3 (1, 2, 3, 25)	2 (1, 2, 3, 25)	3 (1, 2, 4, 9)	0.16
Length of hospital stay > 3 days, n (%)		6 (14.0%)	15 (19.2%)	22 (26.2%)	0.095
MIC, n (%)		23 (54.8%)	41 (54.7%)	45 (53.6%)	0.89

**BMI =** body mass index; **POD =** postoperative day; **eGFR =** estimated glomerular filtration rate; **MIC =** margins, ischemia, and complications.

Data are given as the median (minimum, 25^th^ percentile, 75^th^ percentile, maximum) or number (percent). The change in eGFR from preoperative to POD 1 was reported as the fraction (percent) of patients with an eGFR≤60 at POD 1 among only those who had a preoperative eGFR>60. P values result from the Spearman rank correlation test or the Cochran-Armitage trend test. P values ≤ 0.0042 were considered statistically significant after applying a Bonferroni adjustment.

Comparisons of perioperative outcomes between patients with a low MAP score (0-3) and patients with a high MAP score (4-5) are presented in Table-4. Patients with a high MAP score had longer operative times compared to patients with a low MAP score (median, 213 vs. 192 minutes; P=0.002). No other associations between presence of APF and perioperative outcomes were statistically significant after adjustment for multiple comparisons and there were no other notable associations (all P ≥0.058).

**Table 4:** Perioperative Outcomes according to the Mayo Adhesive Probability Score.

Perioperative outcome		MAP 0-3 (n=145)	MAP 4-5 (n=59)	P value
Operative time, min		192 (106, 170, 216, 320)	213 (135, 183, 228, 336)	0.002
Warm ischemia time, min		19 (0, 14, 23, 42)	19 (0, 14, 24, 35)	0.68
Conversion to open/laparoscopic nephrectomy, n (%)		4 (2.8%)	1 (1.7%)	1.00
Estimated blood loss (mL)		300 (100, 300, 500, 1500)	400 (100, 300, 500, 1200)	0.45
Any postoperative complication, grade I-V, n (%)		32 (22.1%)	12 (20.3%)	0.85
Postoperative complication, grade III-V, n (%)		13 (9.0%)	1 (1.7%)	0.072
**Change in laboratory measures (preoperative to POD 1)**				
	Hemoglobin, mg/dL	−2.1 (−11.2, −2.8, −1.6, −0.2)	−2.2 (−5.5, −3.1, −1.6, 0.0)	0.68
	Creatinine, mg/dL	0.1 (−0.3, 0.0, 0.3, 1.2)	0.2 (−0.3, 0.1, 0.4, 0.6)	0.058
	eGFR≤60, n (%)	45/121 (37.2%)	26/49 (53.1%)	0.062
Length of hospital stay, days		3 (1, 2, 3, 25)	2 (1, 2, 3, 7)	0.77
Length of hospital stay > 3 days, n (%)		28 (19.3%)	14 (23.7%)	0.57
MIC, n (%)		76 (53.5%)	32 (55.2%)	0.88

**MAP =** Mayo adhesive probability score; **POD =** postoperative day; **eGFR =** estimated glomerular filtration rate; **MIC**, margins, ischemia, and complications.

Mayo adhesive probability score was not available for 1 patient. Data are given as the median (minimum, 25^th^ percentile, 75^th^ percentile, maximum) or number (percent). The change in eGFR from preoperative to POD 1 was reported as the fraction (percent) of patients with an eGFR<60 at POD 1 among only those who had a preoperative eGFR>60. P values result from the Wilcoxon rank-sum test or the Fisher's exact test. P values ≤ 0.0042 were considered statistically significant after applying a Bonferroni adjustment.

## DISCUSSION

RAPN is the most common minimally invasive technique currently utilized for nephron sparing in patients with small renal masses (12). The ability to predict which patients are at increased risk of complications and identifying risk factors remains crucial. Additionally, as the prevalence of overweight and obese patients continues to increase, determining how this increased adiposity affects surgical outcomes has become an important interest amongst surgeons. APF is one patient factor associated with adverse perioperative outcomes that can be predicted pre-operatively by radiographic and clinical variables but a full understanding of its pathophysiology remains elusive (8).

We sought to evaluate if surgeon experience eliminated the adverse outcomes associated with the presence of APF. Our study found that even after elimination of the surgical learning curve (100 cases in this study), presence of APF remained associated with longer operative time, suggesting surgeon expertise does not provide the ability to overcome the difficulties associated with tumor dissection and isolation presented by APF. The outcomes in this study are similar to those found in our initial evaluation of the association of RAPN on perioperative outcomes (2). The results of this analysis and our original analysis do not reflect other literature which has found an increase in EBL in patients with APF undergoing RAPN (13). As such, this information could play a vital role not only in patient counseling pre-operatively but also in setting patient and surgeon expectations. There may also be a role for placing APF as a modifier to signify increased surgical difficulty in patients undergoing RAPN if surgeon experience does not eliminate the variation in operative time associated with the procedure.

The incidence of APF in previous publications has been noted to be between 10.6 to 55.2% (13) and the true incidence likely falls somewhere near the middle of these, with the finding of just over 28% in our patient population likely reflecting this. This finding is consistent from our initial analysis of APF (30% in the initial study).

When analyzing the perioperative outcomes between patients with low (≤3) or a high (>3) MAP score, the same outcome of increased operative time was found in both the APF and high MAP score group. The MAP score is a validated scoring system that can be used to predict the presence of APF (4-6, 8, 14). Interestingly, the median operative time was equivalent between patients with a low MAP score and no APF (192 minutes, each), and also those with a high MAP score and APF (213 minutes, each). This again supports the efficacy of the MAP score to predict APF and its utility for surgeons.

Although it can be a risk factor for APF, BMI in itself does not appear to be essential in determining operative difficulty, or at least, surgical outcomes, as supported by our lack of association between BMI and perioperative outcomes of RAPN.

Our study is not without limitations. APF remains a relatively subjective definition. Although some have attempted to define this more objectively (5, 15), a single objective definition of APF may be useful for standardization of future research for determining outcomes. Similarly, our study is based on the experience of one surgeon at a high volume institution for RAPN and may not be generalizable to all surgeons or facilities. Our prevalence of overweight and obese patients was high at 79%. Although this is close to the national average in the United States (71.2% of the population is overweight or obese), worldwide the rate is much lower (39% of adults are overweight and 13% are obese) (16, 17). Given that our study was conducted at a tertiary referral center in the United States, we believe this patient variable is indicative of a more complex cohort and may not be applicable worldwide. Our study sought to evaluate the effect of APF during RAPN after elimination of the learning curve. The learning curve for RAPN has been shown to be well below 100 cases for experienced surgeons. We chose the generous, though arguably arbitrary number of 100 RAPN, for our decided learning curve benchmark. As this study found no significant difference in perioperative outcomes from prior studies and no improvement from our own prior work examining outcomes associated with APF, we cannot say with complete certainty the learning curve of surgically managing APF was eliminated, though we feel most experienced surgeons would agree with our benchmark. In addition, we tried to account for all variables that could possibly be significant to outcomes, but there may be other unaccountable factors that could serve as confounders to our results.

## CONCLUSIONS

Regardless of surgeon experience and proficiency in RAPN, the presence of APF remains associated with longer operative times during RAPN. However, APF does not have an adverse association with other perioperative outcomes after elimination of the surgical learning curve.

## ABBREVIATIONS

RAPN: robotic-assisted partial nephrectomy
APF: adherent perinephric fat
EBL: estimated blood loss
BMI: body mass index
eGFR: estimated glomerular filtration rate
MAP: Mayo adhesive probability
WIT: warm ischemia time
POD: post-operative day
LOS: length of stay
MIC: margins, ischemia, and complications
